# Applications of Hydrogels in Emergency Therapy

**DOI:** 10.3390/gels11040234

**Published:** 2025-03-23

**Authors:** Mariana Chelu, Monica Popa, José María Calderón Moreno

**Affiliations:** “Ilie Murgulescu” Institute of Physical Chemistry, 202 Spl. Independentei, 060021 Bucharest, Romania; calderon@icf.ro

**Keywords:** smart hydrogels, hemostatic materials, drug delivery, emergency care, wound healing, injectable adhesive hydrogel, rapid hemostasis, emergency rescue, antibacterial

## Abstract

Interest in developing new, effective materials for emergency hemostasis and wound healing is steadily increasing, particularly for use in emergency, surgical, and military situations. Hydrogels, with their unique retention, swelling, and biocompatibility properties, have emerged as essential materials in emergency therapy. This review provides a comprehensive examination of recent hydrogel applications in acute medical scenarios, including hemostasis, wound management, drug delivery, soft tissue replacement, and tissue engineering. We discuss the physicochemical properties that make hydrogels suitable for rapid response situations, such as their tunable mechanical strength, adhesiveness, responsiveness to environmental stimuli, and ability to encapsulate and release therapeutic agents. Additionally, the article explores recent advancements in smart hydrogels with self-healing and antimicrobial properties, providing insights into their potential to revolutionize emergency care and increase survival rates in both civilian and military applications. Through a critical evaluation of current clinical trials and practical deployments, this review highlights both the successes and the challenges faced in integrating hydrogels into emergency medical protocols, providing a roadmap for future research and development in this dynamic field.

## 1. Introduction

Effective on-site emergency trauma care requires rescuers to have a solid understanding of fundamental concepts essential for providing first aid to various traumatic injuries. The acute management of trauma, especially major trauma, can follow the protocol described and recommended by the European Guidelines for the Management of Bleeding, developed by a multidisciplinary group of European experts [1].

Severe tissue trauma in emergencies like wars, traffic accidents, or natural catastrophes often leads to uncontrolled hemorrhage, which is a leading cause of mortality worldwide. In most cases, managing hemorrhagic trauma patients with critical injuries demands an urgent, multidisciplinary approach [2,3,4]. One example of a therapeutic emergency is massive hemorrhage [5,6,7]. In such situations, it is essential to quickly identify possible sources of bleeding and promptly adopt the necessary measures to reduce blood loss, restore tissue circulation, and ensure hemodynamic stability [8]. Developing effective hemostatic strategies is vital for saving more military and civilian lives by limiting life-threatening prehospital hemorrhage [9].

**Figure 1:**
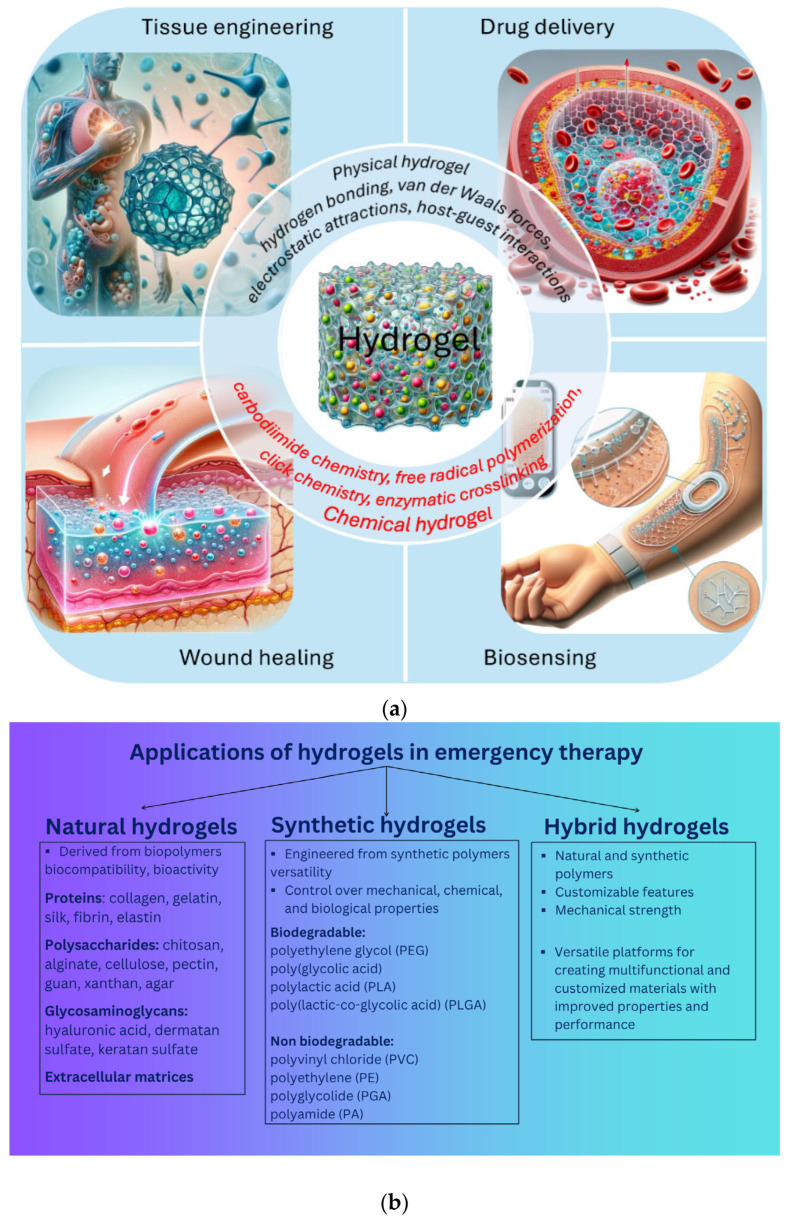
Schematic classification of hydrogels based on crosslinking methods, and biomedical applications (**a**) [15], and different materials (**b**).

Thus, the development of new hemostatic and wound-healing materials remains a pressing challenge, vital for improving the survival rate of patients with critical wounds. The immediate application of emergency hemostatic materials in the initial stage of wound treatment is of crucial importance [10,11,12]. Ideally, the formulation would enable the sealant to be removed without causing further trauma, allowing for gradual wound re-exposure for definitive surgical treatment. However, only a limited number of dressings or sealants meet these requirements, especially the ability to be removed as needed. Hydrogels, with their three-dimensional polymer networks, can retain significant amounts of water, closely resembling human soft tissues and demonstrating excellent biocompatibility [13,14]. They have been widely used in drug delivery, tissue repair, wound closure, and various other biomedical applications (Figure 1) [15,16].

Hydrogels can also serve as scaffolds for tissue engineering, thanks to their porous structures that can house living cells and release growth factors as they dissolve or degrade [17,18]. The main advantage of using hydrogels in wound care lies in their customizable fabrication and characteristics, allowing them to be tailored to specific needs, from the use of natural polymers or the incorporation of nanoparticles and therapeutic biomolecules to the achievement of responsiveness to various stimuli [19]. Thanks to its structure, which closely resembles the extracellular matrix (ECM), and its remarkable capacity to absorb and retain water, hydrogel has become a valuable material in various medical applications. One of its most significant uses is tissue engineering, where it serves as a scaffold that supports cell growth, proliferation, and differentiation, facilitating the regeneration of damaged or lost tissues [20]. Additionally, hydrogels play a crucial role in the production of contact lenses, providing optimal moisture retention and oxygen permeability, which enhances comfort and eye health [21,22].

Moreover, hydrogels are widely employed in wound dressings due to their ability to maintain a moist environment, promote healing, and protect the affected area from infections [23,24,25]. Their capacity to absorb exudates while allowing for gas exchange makes them particularly effective in treating chronic wounds, burns, and ulcers. Another important application of hydrogels is in the controlled release of therapeutic agents, such as drugs, proteins, and growth factors. Their porous structure allows for the gradual and sustained release of medications, improving treatment efficacy and reducing side effects.

**Figure 2:**
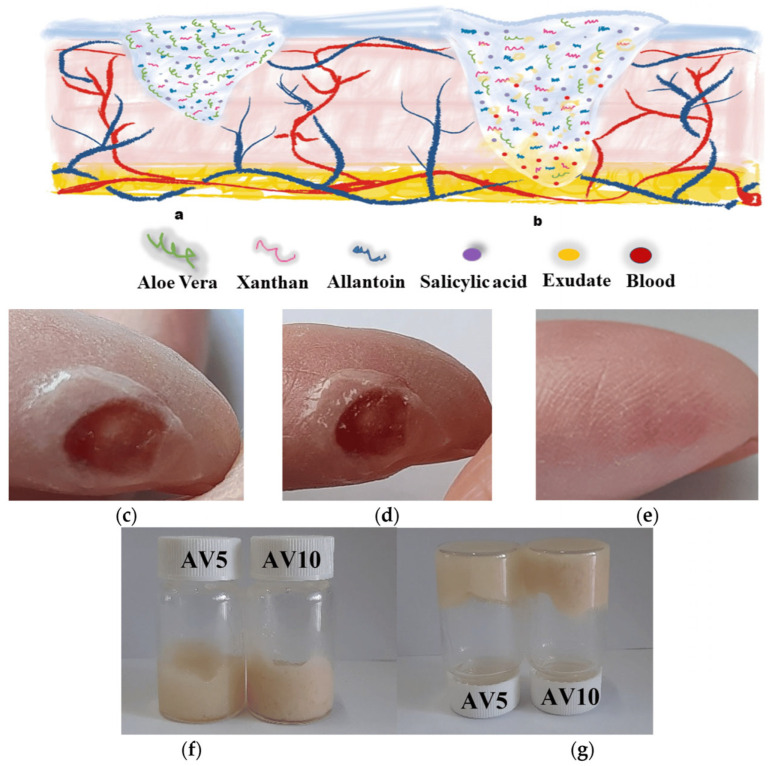
Schematic representation of aloe vera-based hydrogel and its application capacity in wound healing: dry and wet (**a**,**b**); images of the application of aloe vera hydrogel to visualize the progress of wound healing (**c**–**e**); photographs illustrating the hydrogel formation measured using the inverted vial method (**f**,**g**) [26].

Due to these unique properties, hydrogels continue to be at the forefront of biomedical research and innovation, offering promising solutions for regenerative medicine, drug delivery, and advanced medical treatments. Furthermore, the transparency of most hydrogels allows for the monitoring of wound-healing progress (Figure 2) [26].

Although numerous hemostatic hydrogels have been developed using natural and synthetic polymers, only a few are suitable as first-aid treatments for life-threatening hemorrhages in emergency circumstances [27,28]. A major challenge remains ensuring strong adhesion in wet and dynamic conditions. Consequently, there is an urgent need for advanced hydrogel-based hemostatic materials and strategies that can effectively meet the demands of emergency hemostasis [29].

Key areas of concern in trauma cases include injuries related to mechanical forces, electrical exposure, and pressure effects [30]. Trauma can generally be classified into four main categories: penetrating, blunt, deceleration, and thermal injuries [31]. Penetrating injuries involve wounds caused by objects piercing the body, such as stabbings, gunshot wounds, or other foreign bodies [32]. Blunt and deceleration injuries result from impacts, including falls, motor vehicle collisions, or being struck by projectiles [33,34]. Lastly, thermal injuries encompass harm caused by extreme temperatures or substances, including fire burns, inhalation injuries, chemical burns, frostbite, and electrical burns [35,36].

Many traumatic injuries can also be followed by bleeding [37,38]. This can include both external and internal bleeding and can range in severity from minor to massive bleeding [39,40]. Hemorrhage is the primary concern in most trauma cases [41,42]. The management of hemorrhage is guided by its source and severity. Hemorrhage can range from moderate to severe, and therefore the effective initial treatment of hemorrhage is a critical aspect of emergency trauma care [43]. According to Advanced Trauma Life Support guidelines, hemorrhage is categorized into Classes I through IV, with Class IV representing the most severe cases that demand the most intensive treatment. The extent of blood loss dictates the approach to fluid resuscitation and the administration of blood products, such as red blood cells, fresh frozen plasma, and platelets [44]. Blood, which consists of plasma and blood cells, continuously circulates in the cardiovascular system to deliver oxygen and nutrients to tissues [45]. The total blood volume in humans remains relatively stable, a critical factor for maintaining normal physiological functions [46]. Excessive blood loss can lead to severe consequences, as it disrupts normal circulation, impairs microcirculation, and results in hypoxia in the brain and other organs [47]. Uncontrolled bleeding can lead to a rapid decline in a patient’s condition, potentially resulting in complications such as hemorrhagic shock, hypothermia, or metabolic acidosis [48,49]. These conditions can disrupt the usual work of the coagulation system, making it challenging to achieve effective hemostasis. Severe hemorrhage and non-compressible internal injuries are major contributors to high prehospital mortality in both battlefield and civilian trauma cases [50,51]. In addition, uncontrolled bleeding is a leading cause of mortality in cardiovascular, spinal, liver, and orthopedic surgeries [52,53].

Conditions such as blood clotting disorders, including hemophilia, further complicate the process of achieving hemostasis [54].

**Figure 3:**
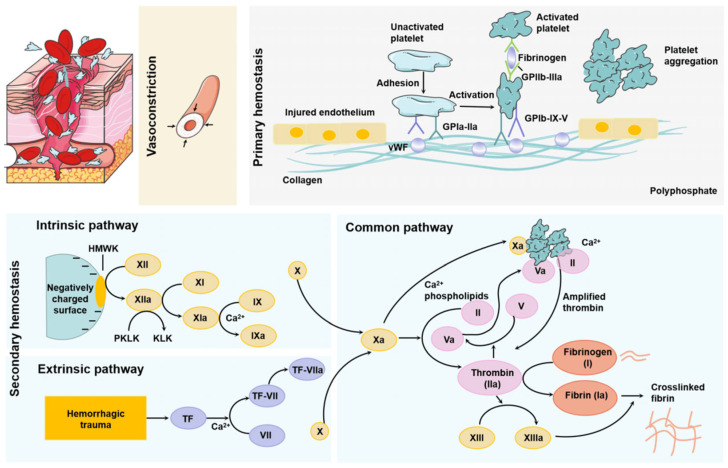
Diagram illustrating the mechanism of physiological hemostasis [58].

When bleeding takes place, the body initiates hemostasis as its natural response. The term “coagulation” is sometimes used in place of “hemostasis”, but this actually refers to only one part of the hemostatic process [55]. Hemostasis in vivo consists of three key processes [56,57]. The mechanism of hemostasis is illustrated in Figure 3 and represents a complex and well-coordinated process by which the body prevents and stops bleeding, maintaining the integrity of blood vessels [58].

During primary hemostasis, platelets interact with collagen, von Willebrand factor, plasma agglutinins, and other endothelial elements to form a platelet clot. Secondary hemostasis involves a coagulation cascade, where prothrombin is converted to thrombin, and fibrin clot formation is regulated [59,60]. Lastly, fibrinolysis helps control clot formation, aiding in the restoration of vascular permeability and normal blood flow [61,62].

In cases of severe bleeding, the natural hemostasis process is insufficient to manage blood loss, necessitating the use of effective external hemostatic methods [63,64]. Consequently, additional interventions are essential to quickly stop bleeding during medical treatment [65,66,67]. Traditional methods like suturing and stapling are considered common practices for wound closure and hemostasis in clinical settings. However, they are not adequate for on-the-spot emergency trauma care due to the need for preoperative anesthesia, laborious protocol steps, and the risk of secondary tissue injury [68]. Numerous recent studies have focused on developing innovative hemostatic adhesives [69,70,71].

Among these adhesives, polymeric hydrogels have gained attention as effective potential hemostats for controlling bleeding and enhancing wound healing, thanks to their remarkable structural and mechanical resemblance to the extracellular matrix (ECM) of biological tissues [72,73,74,75].

**Figure 4:**
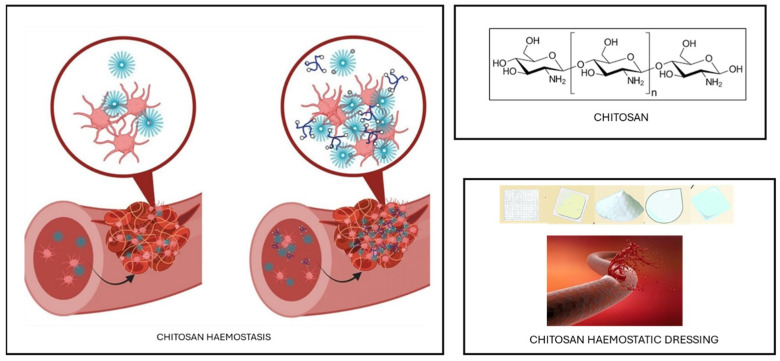
Chitosan-based hemostatic dressings [76].

Natural polysaccharides are excellent materials for medical applications, particularly in hydrogels as wound dressings, as they promote hemostasis and exhibit high biocompatibility with biological tissues (Figure 4) [76,77]. Their effectiveness makes them ideal for managing wounds both before and after surgery.

## 2. Characteristics of Hydrogels for Emergency Trauma Management

An effective hemostatic agent for emergency trauma involving severe bleeding should firmly adhere to tissue, provide a strong and flexible seal, adapt to various complex wound types, and exhibit excellent pro-coagulant and antibacterial properties. It should also be easy to store and administer.

*Key physico-chemical parameters and properties of hydrogels* [74,77]:

- Swelling Behavior—Determines the hydrogel’s ability to absorb water, influencing cell viability, nutrient diffusion, and mechanical stability.
- Mechanical Properties—Include elasticity, stiffness, and strength, which should match the native tissue for proper integration and function.
- Biocompatibility—Ensures that the hydrogel does not trigger an immune response or toxicity, promoting cell attachment and proliferation.
- Biodegradability—Allows for controlled degradation at a rate compatible with tissue regeneration while avoiding harmful byproducts.
- Porosity and Permeability—Affect cell migration, nutrient transport, and waste removal, crucial for sustaining cell activity.
- Crosslinking Density—Impacts mechanical strength, swelling capacity, and degradation rate, influencing overall hydrogel performance.
- Gelation Mechanism and Kinetics—Should allow for easy handling, in situ gelation, and adaptability to different applications (e.g., injectable hydrogels).
- Surface Chemistry—Can be modified to enhance cell adhesion, drug loading, and interaction with biological molecules.
- Hydrophilicity/Hydrophobicity Balance—Regulates water retention, protein adsorption, and cellular response.
- Drug and Growth Factor Delivery Capability—Supports the controlled and sustained release of bioactive molecules for tissue regeneration.

**Figure 5:**
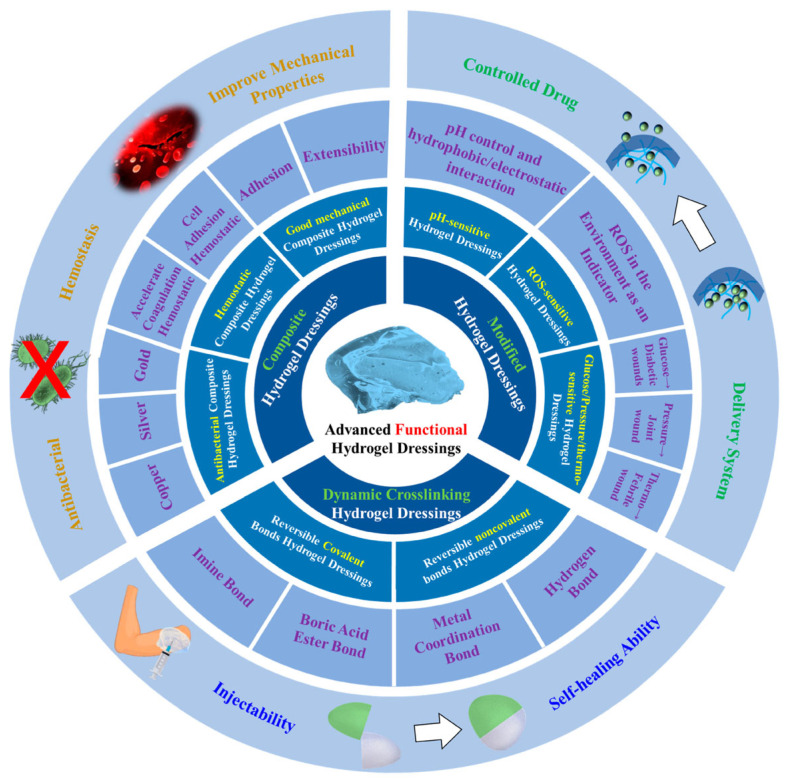
The main characteristics of hydrogel dressings [78].

These properties collectively determine the suitability of hydrogels as effective materials in various biomedical application areas, such as the following (Figure 5) [78,79,80,81,82]:

- Dressings with the release of bioactive substances for wound healing, encompassing areas such as infection prevention, rapid hemostasis and adhesion adaptation, inflammation control and immune regulation, granulation tissue formation, re-epithelialization, and scar prevention and treatment;
- Tissue engineering (3D scaffold, tissue development, growth factor, implantation);
- Controlled drug delivery (in the form of nanocapsules, nanospheres, nanoshells, micelles, niosomes, nanoparticles, dendrimers, liposomes);
- Contact lenses;
- Disposable diapers;
- Cosmetics;
- Biodetection.

Furthermore, the *optimal hemostatic hydrogels* for managing prehospital hemorrhage in emergency circumstances should have some key characteristics, such as the following [72]:

(i) Strong adhesion in wet conditions, high mechanical strength, and suitable swelling properties to quickly halt bleeding and maintain biological pressure such as blood flow and tissue compression;
(ii) Potent antibacterial and wound-healing properties to prevent infections and support tissue regeneration;
(iii) Excellent biocompatibility and biodegradability, ensuring no inflammatory or toxic effects;
(iv) The ability to be quickly deployed and applied.

For *injectable hydrogels*, essential features include the following:

(i) Rapid in situ gelation;
(ii) The ability to be injected for soft filling and in an adaptable manner to irregular wounds, with a ready-to-use format (e.g., prepacked in a syringe without requiring additional preparation);
(iii) Ease of application, allowing use by injured soldiers or untrained civilians.

Among the typical advantages and limitations of different forms of materials based on polymer hydrogels, a few can be listed that highlight their multifunctionality [83,84,85].

Pros and Cons of Hydrogels as Tissue Engineering Matrices:

*Advantages*:

(i) Provide a hydrated environment that safeguards cells and sensitive biomolecules (such as peptides, proteins, DNA, and oligonucleotides).
(ii) Enable efficient nutrient exchange and waste removal, supporting cell viability.
(iii) Can be functionalized with cell adhesion ligands to enhance biocompatibility and interaction with tissues.
(iv) Injectable formulations allow for in situ gelation at body temperature, facilitating minimally invasive applications.
(v) Generally biocompatible, reducing the risk of immune response or toxicity.

*Disadvantages*:

(i) Can be difficult to manipulate and apply in clinical settings.
(ii) Often exhibit weak mechanical properties, limiting their structural integrity.
(iii) Loading drugs and cells while achieving effective crosslinking can be challenging.
(iv) May be unsuitable as prefabricated matrices for in vitro applications.
(v) Sterilization processes can be complex and may alter hydrogel properties.

The great diversity of hydrogel-based materials that have been developed in contemporary research for specific applications in the medical area inevitably leads to the need for careful selection. Depending on the main characteristics and properties presented below, researchers aim to improve the efficiency of clinical translation, from which the majority of patients will benefit [86,87,88].

### 2.1. Robust Mechanical Strength

**Figure 6:**
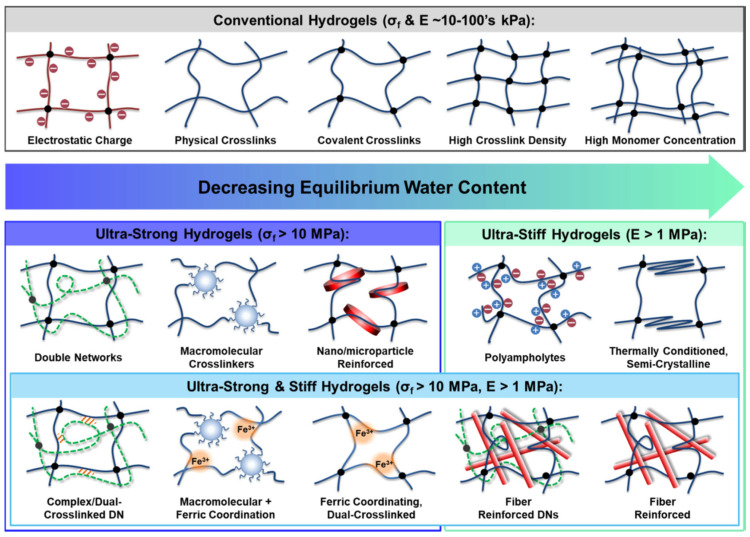
Methods for improving the mechanical properties of hydrogels, classified by fracture strength (σ_f_), modulus (E), and reducing equilibrium water content [89]. Copyright © 2019, American Chemical Society.

The usefulness of hydrogels is constrained by their weak mechanical properties, particularly their insufficient strength and stiffness, which fail to replicate those of tissues, especially load-bearing ones. The mechanical properties of conventional hydrogels are influenced by various determinants, including crosslinking type and density, monomer concentration, homopolymer characteristics (electrostatic charge, hydrophilicity, backbone changeableness), and copolymerization (Figure 6) [89].

Most hydrogels have strengths and moduli in the kilopascal range, comparable to soft tissues like blood vessels, skin, and muscle, because of their high equilibrium water content [90]. The high level of tissue-like hydration enhances the biocompatibility of hydrogels, making them widely usable as safe biomaterials that can interact with biological tissues [91,92,93,94].

For some applications, like replacing load-bearing tissues such as cartilage, tendons, and ligaments, hydrogels with high fracture strengths (σ_f_) and moduli (E) are required [95,96,97,98].

It is well established that increasing crosslinking density and/or monomer concentration directly enhances stiffness [99,100,101]. However, forming a denser polymer network inherently reduces water content and can result in an extremely brittle structure, as the highly stretched polymer chains lack the flexibility to deform before breaking [102,103,104]. As a result, various recent approaches have been explored to improve and tailor the mechanical properties of hydrogels, including interpenetrating networks, macromolecular crosslinking, dual crosslinking, composites, polyampholytes, and thermal conditioning [105,106,107,108].

Hydrogels created using these techniques can typically be classified based on their mechanical properties into three groups: conventional hydrogels, ultrastiff hydrogels, and ultrastrong stiff hydrogels [109,110,111,112,113].

### 2.2. Adhesive Properties

Although various hemostatic agents, sealants, and adhesives possess specific properties, achieving effective hemostasis in wet and dynamic conditions remains a significant challenge [114,115,116]. Adhesive hemostatic agents control bleeding either mechanically or by enhancing the coagulation process and can connect different tissues and blood vessels [117,118,119]. In addition, tissue sealants help prevent blood leakage from vessels. However, many of these products have limitations and are not suitable for all applications. One of the main concerns is related to insufficient strength and durability under physiological conditions, limiting their use in load-bearing applications. In addition, their adhesion capabilities often require improvement to ensure strong and stable integration with biological tissues or synthetic materials [120,121,122].

For wound closure, *hydrogel adhesives* offer significant advantages over sutures and staples, particularly for internal tissue wounds [123,124,125].

Their benefits include:

- High swelling capacity and water content, maintaining a moist environment while absorbing exudate.
- Full wound coverage, ensuring perfect sealing of tissue defects.
- Tunable mechanical properties suitable for subcutaneous tissue, reducing stress concentration at the interface.
- A porous, bioactive structure resembling the native extracellular matrix, fostering a favorable healing environment.
- Functioning as a carrier for cells, drugs, and biological factors.
- Serving as a natural mechanical barrier against infection.
- Biodegradability, eliminating the need for removal.
- Adaptability to various wound types and shapes.
- Being simple and convenient to apply.
- Excellent biocompatibility.
- High customizability for different tissue conditions.

However, it should be noted that achieving strong adhesion under wet and dynamic conditions is a critical challenge when it comes to using hydrogels in medical applications, especially in dynamic and wet environments such as wounds, surgical sites, and tissue interfaces.

The water content in hydrogels disrupts the formation of strong intermolecular interactions (such as hydrogen bonds) between the hydrogel and the surface to which it adheres and reduces the ability of the hydrogel to form stable bonds with biological tissues, leading to poor adhesion or detachment under mechanical stress. Thus, the inability to form stable adhesion can lead to the premature dislocation of hydrogel materials, reducing their effectiveness in maintaining a moist environment and in the healing process.

Hydrogels must maintain their adhesion properties under constant mechanical stress, without detaching, tearing, or failing. However, hydrogels, especially those based on natural polymers, can lose their mechanical integrity or cohesion under continuous dynamic forces. This results in poor long-term adhesion, limiting the effectiveness of hydrogels in applications where continuous contact with tissue is required, such as wound dressings or tissue engineering scaffolds. On the other hand, hydrogels with high elasticity can deform under dynamic conditions, reducing surface contact and disrupting the formation of adhesive bonds. Conversely, hydrogels that are too stiff can crack or lose their integrity, failing to adhere effectively to wet or dynamic surfaces. Last but not least, the degradation of hydrogels, especially under wet and dynamic conditions, can affect their ability to maintain strong adhesion over time.

### 2.3. Antibacterial Properties

Surgical site infections contribute to significant postoperative complications, prolonging recovery time, increasing the risk of further medical interventions, and placing a substantial financial burden on healthcare systems due to extended hospital stays and additional treatments [126]. Bioadhesives designed to fill surgical gaps and promote wound healing typically lack intrinsic antibacterial properties, which can leave surgical sites vulnerable to bacterial colonization and increase the risk of postoperative infections. The development of bioadhesive materials capable of seamlessly integrating with tissue while simultaneously exhibiting antibacterial properties has the potential to greatly reduce the incidence of surgical site infections. By providing both structural support and antimicrobial protection, these advanced materials can help to prevent bacterial colonization at surgical sites, promote more effective wound healing, and ultimately lower the risk of postoperative complications [127,128,129,130,131].

### 2.4. Hemorrhage Control and/or Emergency Hemostasis

Tissue injuries resulting from unexpected emergencies such as natural disasters, traffic accidents, and other traumatic incidents often present significant medical challenges. These injuries typically involve acute hemorrhage, extensive tissue defects, a high risk of wound infection, and, in severe cases, tissue necrosis. The rapid and effective management, even at the site of injury, of such events is crucial to prevent further complications and improve patient outcomes [1]. Traditionally, emergency trauma care has relied on a combination of gauze application to control bleeding and the use of sutures or staples to close the wound [2]. Although this conventional approach has been widely practiced, it is associated with several well-documented disadvantages [3]. A major limitation is its ineffectiveness in simultaneously controlling bleeding and preventing infection, which can lead to additional complications such as delayed healing and an increased risk of sepsis [132]. Sometimes, the suturing or stapling process can lead to secondary injury, inflammation, or pain exacerbation. In addition, this method usually requires the presence of trained medical professionals, including surgeons and anesthesiologists, making it less feasible in emergency situations where access to specialized personnel and medical facilities may be limited.

In cases of severe hemorrhage, excessive blood loss from non-compressible wounds, such as those located in the trunk, groin, or intracavitary regions, poses a significant risk of mortality if not treated promptly [132]. Unlike compressible wounds, which can be effectively treated with direct pressure, non-compressible hemorrhages are particularly difficult to control due to their location and the difficulty of applying external pressure. If left untreated or inadequately managed, such uncontrolled bleeding can quickly lead to major complications or even death.

To address this critical issue, the development and application of effective sealing materials are essential. These materials must not only significantly reduce blood loss at the time of application but also ensure that their removal does not inadvertently cause rebleeding, which could further endanger the patient [132]. An ideal hemostatic agent should form an immediate and stable seal over the wound, preventing further bleeding while simultaneously promoting tissue healing and minimizing complications.

Among the various approaches to hemostasis, highly absorbent hydrogel dressings have shown great potential due to their unique properties [132]. When applied to a bleeding wound, these hydrogels have the ability to rapidly absorb fluids and swell, forming a physical barrier that effectively seals the wound site. This barrier not only helps to stop active bleeding by providing mechanical occlusion but also maintains a moist extracellular matrix, which is optimal for wound healing. A hydrated environment promotes cell migration, enhances tissue regeneration, and reduces the likelihood of scab formation, which can otherwise lead to delayed healing and an increased risk of secondary infections.

Furthermore, advanced hydrogel-based hemostatic materials can be designed to incorporate bioactive components, such as antimicrobial agents or pro-coagulant factors, to further enhance their functionality. Such innovations can improve the effectiveness of hemorrhage control while reducing the risk of infection and excessive inflammation. Given their ease of application, biocompatibility, and effectiveness in managing severe bleeding, hydrogels represent a promising solution for emergency trauma care, especially in prehospital and battlefield situations, where rapid hemostasis is essential for survival. Key characteristics of optimal hemostatic materials based on polymer hydrogels are summarized in Table 1 [132]:

Adhesive hydrogels designed for wet and dynamic tissues play a crucial role in biomedical applications, as they must endure cyclic loading, particularly for controlling hemorrhages on curved skin surfaces and heart tissues. Drawing inspiration from the strong adhesive properties of mussels and *Arion subfuscus*, Fan and colleagues developed a novel polyacrylamide–tannic acid–kaolin hydrogel [133]. In particular, kaolin nanoparticles functioned both as a physical crosslinking agent and as an activator of blood clotting factor FXII, enhancing the coagulation process. The fabricated hydrogels exhibited strong tissue adhesion due to the abundant catechol groups on tannic acid and excellent blood clotting ability, granting them high hemostatic efficiency and making them promising candidates for controlling traumatic bleeding [133].

### 2.5. In Situ Gelation Properties

Lately, hydrogels with special properties, such as injectability, have gained attention as promising materials for the effective management of hemorrhage by promoting hemostasis. These hydrogels have the capability to be delivered via a syringe or needle without compromising their structure or functionality. Injectable hydrogels are engineered to flow easily enough to go through a needle while solidifying or retaining their gel-like consistency once delivered to the body [134]. This characteristic is essential for minimally invasive medical approaches, enabling the hydrogel to be directly injected at the target site [135].

An injectable hydrogel contains a precursor polymer solution combined with various bioactive molecules that gel in situ upon injection to create a solid three-dimensional scaffold. Typically, under normal conditions, these hydrogels continue to exist in a liquid state and go through a sol–gel transition at the target site in response to physiological or external stimuli. Injectable hydrogels not only retain the typical properties of traditional hydrogels but also possess additional features that make them highly effective hemostatic materials. Due to their fluid consistency, injectable hydrogels can be easily applied to deep and irregularly shaped wounds [132]. When administered as a solution, they conform to the wound’s shape before solidifying into a gel, creating effective mechanical barriers that help prevent further blood loss and reduce pain. Furthermore, they can serve as carriers for encapsulated drugs, enabling targeted delivery to specific areas to promoting hemostasis or provide other therapeutic benefits. Due to their superior biomimetic properties, various natural polymers have been the preferred choice for developing injectable hydrogels, some of which have shown promising wound-healing capabilities [136]. In addition to specific bioactivities, natural polymers have inherent viscoelastic properties that aid in the injectability of the hydrogel through pseudoplasticity or shear thinning behavior. The viscosity of the hydrogel decreases with an increasing shear rate [137].

On the other hand, temperature-sensitive systems, which rely solely on temperature as the stimulus, are among the gentlest methods, eliminating the need for additional chemical initiators or enzymatic reactions. This results in a simpler and more cost-effective process. Recently, thermosensitive hydrogels have gained attention as promising drug carriers due to advantageous characteristics such as high drug loading capacity, site-specific targeting, and the ability to provide sustained drug release, biodegradability, and low toxicity [138].

**Figure 7:**
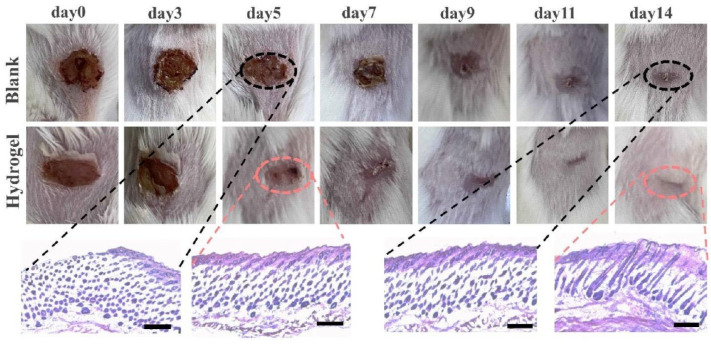
Images showing wound healing in mice using the optimized hydrogel for 14 days compared to a control group, with H&E staining on days 5 and 14 (scale bar of 200 μm) [72]. Copyright © 2023, American Chemical Society.

All these features have led to advances in thermoresponsive hydrogels. Song H et al. (2023) developed a multifunctional hemostatic biopolymer-based hydrogel with extracellular matrix, which simultaneously integrated rapid thermosensitive gelation, robust wet adhesion, and ease of use in emergency situations [72]. In this context, four functionalized components were synthesized based on the backbone of gelatin (G) and hyaluronic acid (HA) for hydrogel preparation: poly-N-isopropylacrylamide-grafted gelatin (G-P), quaternary ammonium-modified gelatin (G-Q), poly-N-isopropylacrylamide-grafted HA (HA-P), and o-nitrobenzyl (NB)-modified HA (HA-NB). These modifications were introduced to impart thermosensitivity, antibacterial properties, and blood coagulation capabilities to the hydrogels. The performances of the synthesized hydrogels were evaluated and optimized in terms of adhesion and compressive strength, burst pressure, and swelling ratio, as well as rheological, antibacterial, and cytocompatibility properties. The optimal hydrogel, named G-P15/HA-P1/G-Q1/HA-NB0.4, was prepared by a photo-crosslinking reaction and exhibited rapid thermosensitive gelation and robust wet adhesion. The hydrogel can be easily applied through simple injection, undergoing an instantaneous sol–gel phase transition at body temperature (Figure 7) [72].

## 3. Applications of Hydrogels in Emergency Therapy

As discussed in the introductory section, a traumatic injury refers to a physical injury that occurs suddenly and with significant severity, often resulting from external forces such as accidents, falls, or violent impacts. These injuries typically require urgent medical intervention to prevent complications, minimize damage, and promote effective recovery. Certain injuries can be promptly treated at the site where they occur. However, the majority of accidents leading to traumatic injuries can be effectively managed in hospital emergency departments, where medical professionals are equipped with the necessary expertise, technology, and resources to provide immediate assessment, stabilization, and treatment. This timely medical intervention helps prevent further complications, reduces the risk of long-term disability, and improves patient outcomes.

The selection of specific hydrogel materials for various emergency applications is a critical decision that depends on several factors, including the intended use, required properties, and the unique challenges posed by the emergency situation. Hydrogels are versatile materials, and their use in emergency settings spans a wide range of applications, from wound care to drug delivery systems. For wound care, hydrogels must possess properties such as high water content, biocompatibility, and the ability to promote healing by maintaining a moist environment, which is essential for speeding up recovery and reducing the risk of infection. Additionally, hydrogels used in such applications must be sterilizable and capable of adhering to the wound site without causing irritation. In drug delivery, hydrogels are chosen for their ability to encapsulate and release therapeutic agents in a controlled manner, which is particularly useful in emergency situations where the rapid or sustained release of medication is necessary. The choice of hydrogel in this case would depend on factors such as biodegradability, the type of drug being delivered, and the required release profile (fast or slow). For trauma care, hydrogels with enhanced mechanical strength and flexibility may be needed, as they could be used to support or stabilize injured tissue. These hydrogels would need to withstand the stresses of being applied to different body areas, and their ability to conform to irregular wound shapes would be critical. Ultimately, the selection process involves balancing these factors to ensure that the hydrogel provides optimal performance in the emergency context, ensuring it is not only effective but also safe and easy to use in high-pressure, time-sensitive environments.

### 3.1. Hydrogels for Ophthalmic Injuries

Among the various high-risk but low-prevalence conditions encountered in the emergency department, ophthalmological emergencies represent a critical category. These conditions, though relatively uncommon, require prompt diagnosis and specialized intervention to prevent potential complications, including vision loss or long-term impairment. Due to the complexity and urgency of such cases, timely evaluation by trained medical professionals is essential to ensure optimal patient outcomes.

Open globe injury is a severe and relatively uncommon ocular trauma that involves a full-thickness wound of the eye wall. This condition is associated with a significant risk of vision loss and long-term complications, often requiring urgent surgical intervention and extensive postoperative care to preserve ocular function and minimize morbidity [139]. Open globe injury can result from either blunt or sharp trauma and encompasses various subtypes, including penetration, intraocular foreign body, perforation, globe rupture, or mixed forms. It is more frequently observed in males, often due to work-related accidents, whereas in females, falls are the leading cause.

For the rapid sealing of open globe lesions, an injectable hydrogel with double thermo/photo-crosslinking was prepared based on arginine and hydroxybutyl chitosan modified with methacrylic anhydride for use in emergency ophthalmic cases [140]. It was obtained in liquid form at ambient temperature to facilitate the injection process at the site of the lesion. Upon contact with the higher-temperature ocular surface, the hydrogel underwent reversible heat-induced gelation to achieve in situ transformation. Subsequently, under UV light, a double-network structure with mechanical strength was obtained through rapid photo-crosslinking.

Burst pressure tests performed ex vivo and in vivo demonstrated the ability of the hydrogel to rapidly restore intraocular pressure and to rapidly and effectively seal open globe lesions [140].

### 3.2. Hydrogels for Organ Injuries

Gastric perforations can be caused by trauma, malignancy, interventional procedures, or intrinsic gastric pathology and represent an immediate emergency in order to avoid complications leading to sepsis [141,142,143]. The repair of stomach trauma remains a critical focus in the biomedical field, as effective wound closure is essential for preventing complications and promoting healing [144,145,146,147]. Although extensive research has been conducted on the development of gastric hydrogel patches, many existing solutions face challenges such as insufficient adhesion in a wet environment or undesirable double-sided adhesion, which can lead to unintended attachment to surrounding tissues [148]. As a result, the design of asymmetric adhesive hydrogels has emerged as a promising approach for the advancement of suture-free wound-sealing patches specifically tailored for stomach trauma repair [149,150].

**Figure 8:**
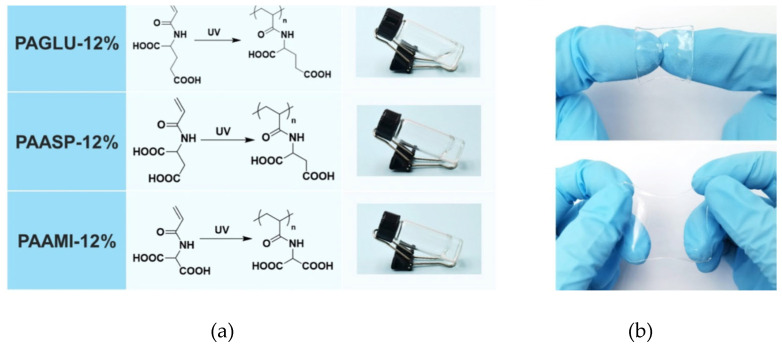
Images of PAGLU-12%, PAASP-12%, and PAAMI-12% hydrogels (**a**) and of PAASP-35% hydrogel in extension (**b**) [151].

For first-aid cases, rescue, and the urgent repair of gastric perforations, adhesive hydrogels were developed by free-radical photopolymerization using different concentrations in water (12–35%) of three vinyl monomers, namely N-acryloyl glutamic acid, N-acryloyl aspartic acid, and N-acryloyl amidomalonic acid (Figure 8) [151].

**Figure 9:**
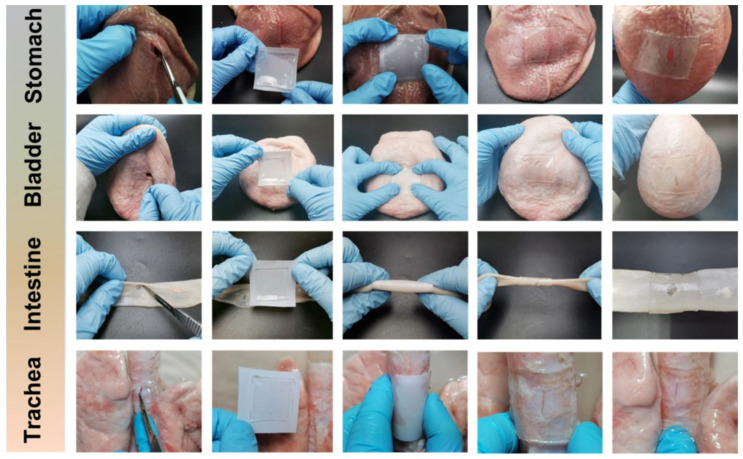
Images showing the strong adhesion of the PAASP-35% hydrogel patch, which seals an incision of approx. 5 mm, to various ex vivo porcine organs (stomach, bladder, intestine, and trachea) [151].

Among them, the PAASP-35% hydrogel obtained by free-radical polymerization of N-acryloyl aspartic acid proved to be a strong adhesive. It showed remarkable mechanical properties, such as good strength, robust elasticity, and excellent flexibility, as well as strong adhesion to biological tissues. To prevent peritoneal adhesion during in vivo tissue repair experiments using the PAASP-35% hydrogel patch, a Janus hydrogel patch was developed through Fe^3+^ transfer printing on paper. Featuring both adhesive and non-adhesive surfaces, this patch was applied in the treatment of gastric perforation in mice. The results demonstrated that hydrogel effectively promoted tissue repair while preventing postoperative adhesion. The potential of PAASP hydrogel for rapid wound closure was assessed through an ex vivo assay using various porcine organ models. After making incisions in various organs, including the stomach, urinary bladder, small intestine, and trachea, PAASP-35% hydrogel was applied as a tissue sealant, along with a polytetrafluoroethylene (PTFE) film attached on the back, to close the wounds (Figure 9) [151].

**Figure 10:**
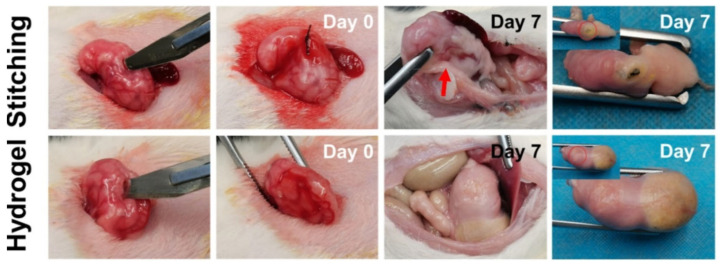
Images of Janus patch treatment for an approximately 5 mm incision in a mouse gastric perforation model and the healing effect after 7 days. In the images, the red arrow in the suture group marks the site of postoperative adhesions [151].

Qualitative assessments revealed that the hydrogel adhered securely to the organ surfaces, effectively preventing any leakage. This strong adhesion was attributed to its remarkable flexibility and adhesive properties. Furthermore, quantitative tests were conducted to evaluate the hydrogel’s stability and adhesion strength to biological tissues following immersion in water. In Figure 10, the Janus hydrogel prepared by an innovative filter paper transfer printing method to achieve single-sided patterning with Fe³⁺ is presented. The findings revealed reduced adhesiveness and the effective prevention of abdominal adhesions. These results confirm that the Janus hydrogel enables selective adhesion, making it suitable for in vivo applications [151].

The rapid formation of an adaptable and durable biointerface between bioadhesives and intricate tissue surfaces is a crucial characteristic of high-performance tissue adhesives. However, achieving effective tissue regeneration remains challenging due to the wet and dynamic nature of complex physiological environments. Typically, high mechanical strength and strong adhesive capacity are difficult to achieve simultaneously. Most hydrogels with physical crosslinks must compromise mechanical performance to enhance adhesion or sacrifice adhesion for greater strength, limiting their broader application. Thus, designing hydrogel materials that simultaneously achieve outstanding adhesion and mechanical strength is essential for expanding their practical applications.

Drawing inspiration from mussel adhesion, the incorporation of catechol-based molecules facilitates the formation of catch bonds in moist environments. Additionally, these molecules induce a crowding effect by forming biomolecular conglomerates, enhancing strong underwater adhesion.

**Figure 11:**
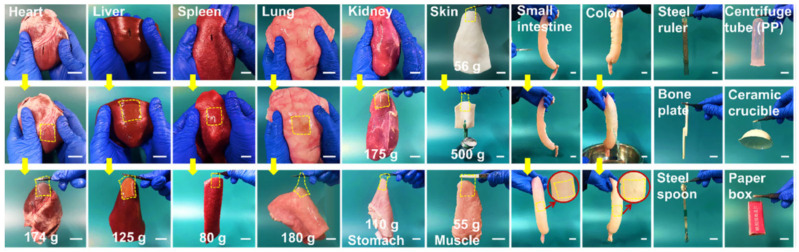
Digital images of the adhesion performance of hydrogels prepared using different porcine organs. Yellow arrows mark the edges of the hydrogel (at a scale bar of 20 mm) [152].

To prepare supramolecular hydrogel patches for rapid hemostatic intervention and effective wound closure with good adhesion and mechanical capacities and multiple biofunctions, a hierarchical multi-strength hydrogen-bonding strategy can be employed [152]. This approach constructs a polyacrylic acid-based hydrogel network through a one-pot UV-induced copolymerization process, incorporating epigallocatechin gallate molecules via physical entrapment. The engineered supramolecular hydrogel patch, endowed with bioactive properties, exhibits remarkable mechanical properties (e.g., stretchability, tensile strength, ultra-high toughness, instant and robust tissue adhesion, endurance under cyclic loading), reversible adhesion, and excellent anti-swelling performance. The obtained supramolecular hydrogel patch achieves rapid hemorrhage control (60 s) in liver injury and promotes effective wound closure and healing in full-thickness skin incisions, reducing inflammation and minimizing scarring. Figure 11 shows photographs of the hydrogel adhering to various substrates and closing artificial wounds in porcine organs while supporting its own weight [152].

**Figure 12:**
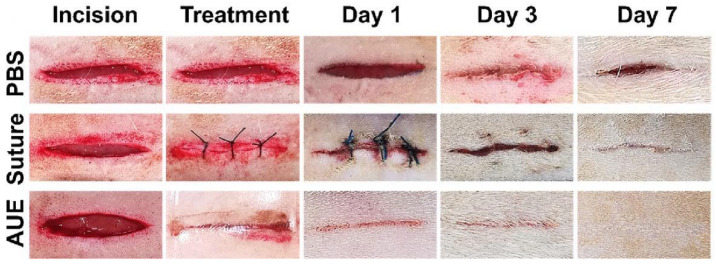
Photographs of in vivo wound healing observed for 7 days after treatment with the prepared AUE hydrogel compared to suture and phosphate buffered saline (PBS) [152].

In an in vitro experiment, the wound-healing process was monitored for seven days from the time of incision (Figure 12) [152]. The transparent feature of the hydrogel facilitated the observation of the wound condition and the avoidance of secondary effects. On the last day, as shown in the images, it was observed that the wound surface closed continuously and uniformly, with imperceptible scars. These findings highlight the potential of this material as an easy-to-use self-rescue adhesive patch for vulnerable individuals to prevent hemorrhage and close wounds without sutures.

Controlling excessive bleeding is essential to prevent life-threatening complications in traumatic or surgical injuries. In recent years, significant efforts have been made to develop adhesives for wound closure and hemostasis. Injectable adhesives, which can form a gel in situ when triggered by various stimuli, are particularly promising for tissue wound closure. These injectable adhesives are effective for filling deep or irregularly shaped wound defects and also provide a physical barrier against bacterial microorganisms. However, due to their slow gelation rate, poor adhesion to moist tissues, and inferior mechanical properties, these adhesives are not recommended for the treatment of large, bleeding wounds.

**Figure 13:**
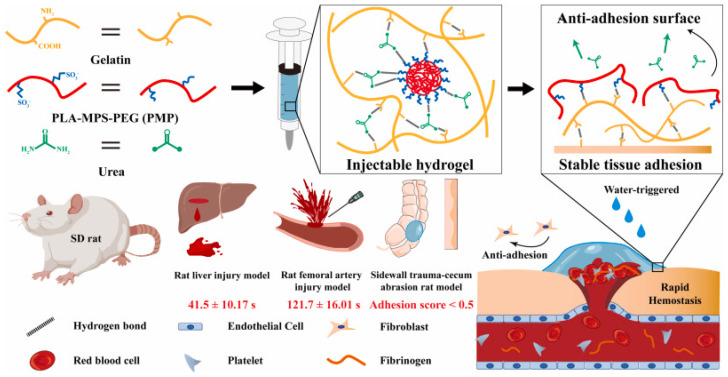
A schematic illustration of the PMP–gelatin hydrogel, showcasing its capability to conform to irregular wound geometries, ensure stable adhesion, and promote rapid hemostasis. Upon water treatment, the hydrogel surface transforms into a hydrophobic barrier, effectively preventing adhesion [153].

In a recently presented study, an injectable hydrogel consisting of hydrophobic polylactic acid modified with hydrophilic polyethylene glycol and antiprotease zwitterionic sulfobetaine, to which urea and gelatin were added, was prepared [153]. The goal of this study was to develop a synthesized material that, upon contact with water, forms a smooth film structure, effectively covering irregular bleeding areas and accelerating wound closure while providing anti-adhesive properties (Figure 13) [153].

**Figure 14:**
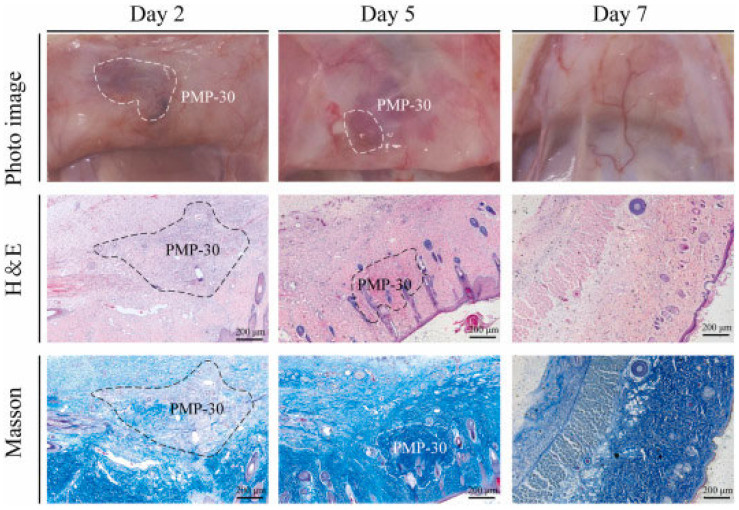
Macroscopic images, along with H&E and Masson trichrome staining, of the implanted PMP-30 hydrogel in rat dorsal subcutaneous tissue. The area outlined by the dashed line indicates the residual PMP-30 hydrogel (at a scale bar of 200 μm) [153].

Through experiments carried out both in vivo and in vitro, biocompatible properties, tissue adhesion, rapid response to the hydrophobic interface, biodegradability, and rapid hemostasis for incompressible wounds were demonstrated. In addition, the effectiveness of the hydrogel in the case of incompressible bleeding was explored, and it was shown that it could prevent subsequent tissue adhesion based on the simply triggered anti-adhesion system and the anti-protein properties of zwitterions (Figure 14) [153].

In recent years, many injectable hydrogel adhesives have been developed that can rapidly penetrate various soft tissues and form strong bonds with them, ensuring solid adhesion during dynamic movements. Complex hydrogels, which contain biochemical molecules and natural polymers, offer excellent biocompatibility and stable physicochemical properties. The integration of these natural polymers has significantly improved the stability, mechanical resilience, and adhesive strength of injectable hydrogel adhesives at tissue interfaces. Thus, the development of such natural polymer-based adhesive hydrogels has great potential for clinical applications in the management of uncontrolled bleeding [154].

**Figure 15:**
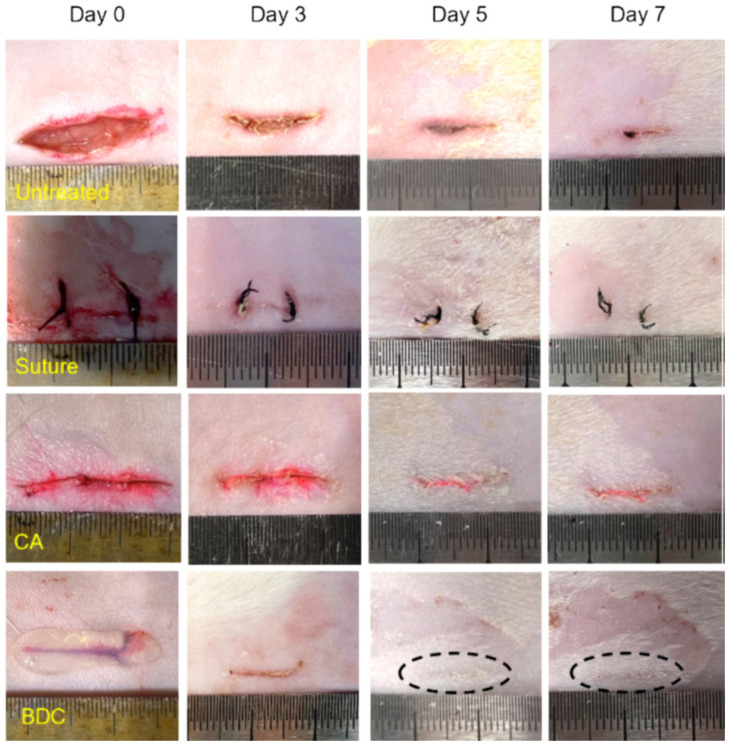
Images of wound healing in rats, monitored for 7 days after treatment with BDC hydrogel compared to blank, suture, and CA glue [154].

Liu T et al. (2024) fabricated a double-crosslinked bionic hydrogel (BDC) of poly(γ-glutamic acid) and poly(N-(2-hydroxyethyl) acrylamide) by combining the photo-initiated radical polymerization technique with the hydrogen-bonding crosslinking method within seconds of ultraviolet (UV) irradiation [154]. The developed hydrogel exhibited an ultrafast gelation process after only one second. In addition, it demonstrated excellent mechanical properties (e.g., strong adhesion to wet pig skin, resistance to ultra-high burst pressure). The ultrafast gelation ability and ultra-high burst pressure tolerance of the BDC hydrogel ensured fast, controllable adhesion that enabled rapid hemostasis. The wound treated with the BDC hydrogel healed faster than those in the control groups, highlighting the potential of this hydrogel in emergency rescue and wound care scenarios. Images of incisions in rats that were treated for seven days are shown in Figure 15 [154].

**Figure 16:**
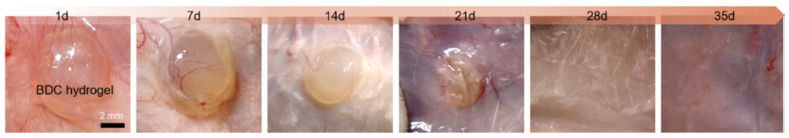
Macroscopic evolution for a period of 35 days of the implanted BDC hydrogel [154].

In vitro and in vivo biocompatibility and biodegradability tests showed that the BDC hydrogel did not exhibit cytotoxic effects. In addition, it was implanted subcutaneously on the back of a rat and observed for a period of 35 days. The images show that the inflammatory response that appeared in the first hours decreases and almost completely disappears after 35 days (Figure 16) [154]. The ability to heal full-thickness skin wounds and deep hepatic injury highlights the potential of the BDC hydrogel as a surgical adhesive for hemostasis, wound closure, and healing, including irregular and dynamic moist tissue surfaces.

To explore new treatment strategies for intrauterine adhesions, in a very recent study, researchers utilized self-healing conductive hydrogels, which mimic the physiological environment of the extracellular matrix and serve as effective physical scaffolds within the uterine cavity [155].

A new injectable hydrogel composed of chitosan–lignin and poloxamer loaded with platelet-rich plasma (CL-PF127@PRP) was successfully developed through self-assembly at room temperature. In vitro cell experiments confirmed that the hydrogel exhibited excellent biocompatibility, anti-inflammatory properties, and pro-angiogenic activity. Furthermore, animal studies on skin wounds and intrauterine adhesions (IUAs) demonstrated that the hydrogel achieved a skin wound closure rate exceeding 50% by the seventh day. Platelet-rich plasma enhanced endometrial thickness and uterine receptivity, indicating that the CL-PF127@PRP hydrogel holds significant potential for the clinical treatment of intrauterine adhesions (IUA) [155].

### 3.3. Hydrogels for Brain Injuries

Traumatic brain injury, resulting from external forces that disrupt brain function or cause detectable brain damage, represents a major global health concern. Brain injuries are among the most severe human conditions, often leading to death or lasting disabilities. They are the primary cause of mortality and long-term impairment worldwide. Additionally, traumatic brain injuries are the leading trigger of seizure disorders. The main causes of traumatic brain injuries are due to motor vehicle accidents (about 50% of all), falls (especially in people over 65 years of age), transportation (people under 65), and sports (skiing and ice skating) [156]. Traumatic brain injuries are difficult to accurately detect and correctly diagnose. In addition, they are associated with numerous disorders, including amnesia, Alzheimer’s and Parkinson’s diseases, sleep disorders, and other impairments in physical, cognitive, and mental functioning [157].

In the treatment of brain injuries due to severe trauma, emergency surgery to remove damaged brain tissue and control intracerebral hemorrhage is a critical priority [158,159].

During the acute phase of traumatic brain injury, the equilibrium between brain oxygen supply and energy sources is disrupted, leading to heightened oxidative stress responses and disturbances in energy metabolism within the microenvironment of the injured brain tissue. Excessive oxidative stress, combined with the buildup of reactive oxygen species, contributes to secondary injury [160,161].

In parallel with other biochemical, molecular, and metabolic disorders, ROS cause mitochondrial dysfunction, which can worsen the condition of patients, even inducing apoptosis. Hence reducing oxidative stress and restoring energy metabolism are crucial in the area of brain injuries. For the treatment of TBI and combating oxidative stress, numerous antioxidant therapies have been investigated [162,163], and various antioxidant and metabolic agents have been developed [164,165,166,167]. However, conventional systemic intravenous drug therapy for postoperative delivery is often ineffective.

Hydrogels are biocompatible, biofunctional materials with sustained drug release and targeted delivery capacity [168]. These advantages have led to studies and applications for various neuropathologies [169,170,171,172]. In the treatment of traumatic brain injury, hydrogels should be strong enough to maintain the local tissue structure while also matching the rheological properties of brain tissue to reduce stimulation of the injured area. As a result, the mechanical and rheological properties of hydrogels play a crucial role in their effectiveness for treating traumatic brain injury [173]. Also, one of the most significant challenges for hydrogel-based treatments in TBI is the highly selective blood–brain barrier, which protects the brain from potentially harmful substances in the bloodstream but also complicates the administration of therapeutic agents. Although hydrogels offer the potential for the controlled release of drugs and bioactive molecules, delivering these agents to the brain is a formidable challenge. In practice, hydrogels have a limited capacity to address the widespread neuronal damage characteristic of TBI. Achieving the correct degradation rate of hydrogels, especially those designed for drug delivery, that aligns with the TBI healing process is also a significant challenge. If hydrogels degrade too quickly, they may not provide sustained release of therapeutic agents, while if they degrade too slowly, they may cause a prolonged local inflammatory response or interfere with tissue regeneration. This can lead to the formation of fibrotic tissue or scar formation, which can exacerbate neurological damage.

**Figure 17:**
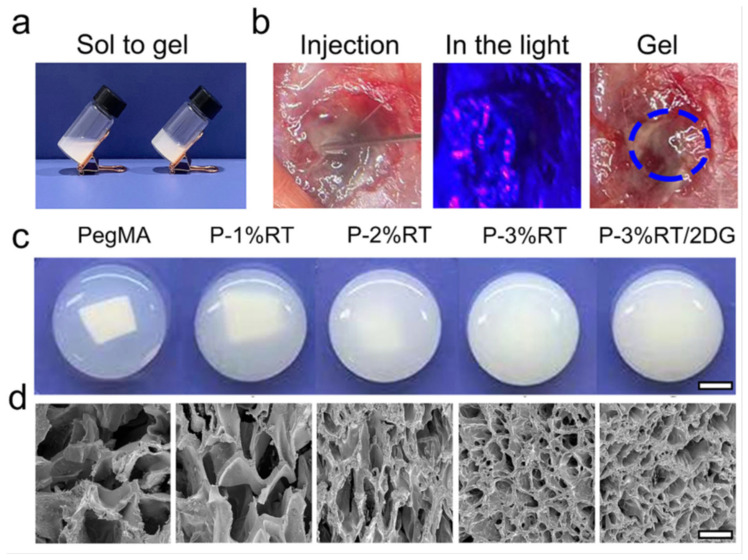
Images of the prepared hydrogel (**a**,**b**). Transition of the hydrogel from sol to gel in vitro and in vivo, (**c**) with different concentrations of RT ranging from 1 to 3%, and (**d**) microstructure confirmed by SEM [160].

A new type of self-assembling smart hydrogels has been developed to correct oxygen and energy metabolism in TBI and can be applied intraoperatively directly to the brain [160]. At the same time, a crosslinking agent of the hydrogel in the form of a thioketal linker (RT) was prepared to respond to ROS. The injectable solution based on a glucose analog, namely 2-deoxyglucose (2DG), together with RT and poly(ethylene glycol) dimethacrylate, self-assembles under the application of blue light. In the microenvironment of brain lesions, due to its antioxidant properties, the hydrogel degrades in the presence of ROS, eliminating them and releasing 2DG. This material was specifically engineered to enhance the hydrogel’s efficiency by ensuring the sustained release of 2DG and precise delivery to the injured areas. Figure 17 [160] shows in vitro and in vivo gelation processes of the hydrogel, before and after exposure to blue light. With the increase in RT concentration (from 1% to 3%), it was observed that the transparency of the hydrogel gradually decreased, a characteristic also confirmed by scanning electron microscopy.

**Figure 18:**
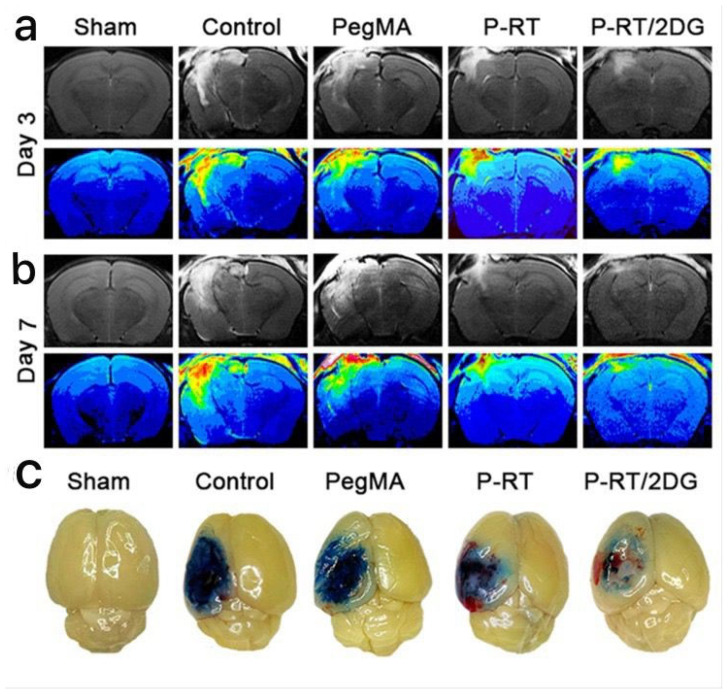
Decreasing cerebral edema and maintaining BBB integrity in the presence of hydrogels. (**a**) Images on the third day after TBI, (**b**) images on the seventh day after TBI, (**c**) images during the use of EB on the seventh day after TBI [160].

In an in vivo experiment performed on mice, the authors tested the developed hydrogels by simulating clinical conditions using a severe controlled cortical impact (CCI) model. In Figure 18 [160], images from the evaluation, performed with the help of magnetic resonance imaging on days 3 and 7 after TBI, are presented. On the seventh day after TBI, blood–brain barrier (BBB) permeability was assessed using Evans blue (EB) extravasation. The observations (Figure 18c) [160] showed a low level of EB penetration, demonstrating that the P-RT/2DG hydrogel showed the best BBB protective function.

### 3.4. Hydrogels for Spinal Cord Injuries

Some of the most dramatic accidents are spinal cord injuries that lead to almost irreparable destruction of both cells and the fiber tracts that carry information in both directions. Unfortunately, after spinal cord injury, there are no effective clinical strategies for axon regeneration, as spinal cord neurons have limited intrinsic regeneration capacities [174]. Numerous experimental strategies are currently being developed with optimism to improve spinal cord reconnection and rewiring for functional recovery. Among the approaches considered, several directions stand out: (i) axonal regeneration (direct endogenous reconnection), (ii) axonal sprouting (indirect endogenous reconnection), and (iii) neuronal stem cell transplantation (indirect exogenous reconnection) [175].

Alternatively, a novel biomimetic 3D injectable hydrogel platform with aligned topography was developed for spinal cord injury repair [174]. Well-aligned fibrils in a 3D matrix were synthesized using biomimetic thermal gelation polymers in a facile and reproducible manner. The thermal gelation (at 37 °C) hydrogel system was fabricated by an aspiration and ejection method, alternatively combining type I collagen with two other native extracellular matrix proteins, laminin I and hyaluronic acid. The materials, which were designed as natural supports to promote axonal growth and the alignment of primary embryonic spinal neurons, were studied in vitro. Following the implantation of the hydrogels in the T7/T8 thoracic spinal cord, host cell and neurite outgrowth was observed. The authors concluded that the in vivo implantation of the fabricated hydrogels integrated well into the host tissue and supported endogenous axonal growth by guiding it. However, further studies are needed to monitor the experimental results regarding an improvement in functional recovery [174].

### 3.5. Hydrogels for Acute Colitis

Inflammatory bowel disease, including Crohn’s disease and ulcerative colitis, is increasingly prevalent worldwide, and current therapies are not completely effective. Patients suffering from inflammatory bowel disease have a tendency for an increased risk of developing colorectal cancer [176,177]. Recently, due to acute painful symptoms, several studies have addressed this emerging problem [178,179].

A novel intrarectal formulation of olsalazine-loaded composite hydrogel microspheres with good bioadhesiveness and reactive oxygen species (ROS) scavenging capacity was developed to enhance drug retention and therapeutic efficacy [180].

Upon intrarectal administration, negatively charged and dopamine-functionalized hydrogel microspheres effectively adhered to the cationic surface of the inflamed mucosa, scavenging ROS and releasing Zn^2+^ and Olsa for antibacterial and anti-inflammatory effects. These active components of the hydrogel microspheres attenuated acute intestinal inflammation in a mouse model of dextran sodium sulfate-induced colitis.

### 3.6. Hydrogels for Localized Drug Delivery

At present, surgical interventions, often complemented by systemic drug delivery, represent the primary treatment approach for a wide range of medical conditions, including cancer and trauma-related injuries. Surgery serves as a major method for removing tumors, repairing damaged tissues, and restoring physiological function, while systemic drug administration—through oral, intravenous, or other routes—ensures targeted therapeutic effects to manage symptoms, prevent complications, and enhance recovery [9]. However, surgery presents several challenges, including pain, inflammation, slow tissue regeneration, the risk of disease recurrence, and non-specific toxicity associated with chemotherapy. These side effects continue to be significant clinical concerns, often complicating recovery and limiting the overall effectiveness of treatment. Hydrogels serve as effective local drug delivery carriers, helping to reduce the side effects associated with conventional systemic drug administration [150]. Their biocompatibility and controlled-release properties make them particularly suitable for treating a wide range of surgery-related injuries, enhancing recovery while minimizing adverse effects [181].

Intervertebral disc degeneration is a leading cause of chronic low back pain and a major contributor to disability worldwide [182]. This degenerative process involves the progressive breakdown of the disc’s structural and functional integrity, leading to reduced shock absorption, inflammation, and nerve compression. Despite various treatment options, including pain management, physical therapy, and surgical interventions, current approaches primarily focus on symptom relief rather than reversing the degenerative process. Due to the lack of regenerative capacity in existing treatments, there remains an urgent need for innovative therapeutic strategies aimed at restoring disc structure and function.

A novel hydrogel formulation based on hyaluronic acid and type II collagen was developed for tissue repair targeting pain in IVD degeneration [182]. The therapeutic effect was evaluated by implanting the prepared material into surgically induced disc lesions at the coccygeal level in a rat tail pain model. Disc hydration and a reduction in pain behavior were observed using magnetic resonance imaging (MRI). The authors propose that the hydrogel suppresses hyperinnervation while enhancing disc hydration, facilitating tissue repair. These properties indicate its potential as a promising therapeutic option for treating degenerative lumbar disc pain [182].

### 3.7. Hydrogels for Breast Cancer Therapy

Breast cancer is the most frequently diagnosed malignancy in women and stands as the second leading cause of cancer-related mortality among women worldwide [183]. The management of breast cancer largely depends on factors such as the tumor’s grade, stage, and molecular characteristics. Treatment typically involves a combination of surgical intervention to remove the tumor, followed by antineoplastic therapies such as chemotherapy, radiation, and targeted therapies. These approaches aim to eliminate remaining cancer cells, prevent recurrence, and improve overall survival rates. The specific treatment plan is tailored to each patient based on the tumor’s behavior and response to previous therapies. However, limitations due to acute or chronic side effects of chemotherapy highlight the importance of minimizing these adverse effects to enhance the overall benefits of these treatments. Hydrogels, biomimetic materials featuring three-dimensional network structures that are both elastic and flexible, have garnered significant attention for their potential to enhance breast cancer therapy [184,185,186]. These materials possess unique properties that allow them to mimic natural tissue environments, providing an ideal medium for controlled cytotoxic drug delivery in the microenvironment of breast cancer and tissue regeneration [187,188,189,190,191,192,193]. Hydrogel implants, in particular, can be engineered with specific properties, such as adipogenic (fat cell-forming) and angiogenic (promoting blood vessel growth) capabilities, to support tissue integration and repair. By fostering a favorable microenvironment for tissue growth and promoting vascularization, these hydrogels can improve the healing process after the surgical resection of breast tumors as post-surgery prosthetic scaffolds while simultaneously delivering chemo/immunotherapeutic agents directly to the affected area [194,195,196,197,198,199,200]. This approach may significantly enhance the effectiveness of treatment and reduce the risk of tumor recurrence. A novel strategy using injectable and thermosensitive hydrogels has been developed to achieve the targeted and prolonged delivery of Herceptin, an antibody used in the treatment of HER2+ breast tumors [201]. The intended goal was to reduce the risk of local recurrence after breast-conserving surgery and to minimize systemic side effects, especially cardiotoxicity. The hydrogels were prepared based on two triblock copolymers poly(lactic acid-co-glycolic acid)-b-poly(ethylene glycol)-b-poly(lactic acid-co-glycolic acid) (PLGA-PEG-PLGA) with different PEG/PLGA ratios of the blend, which were then loaded with Herceptin. The results demonstrated the prolonged release of Herceptin from the hydrogel matrix in vitro for up to 80 days, marking the longest reported delivery period for Herceptin to date. Studies on the in vivo behavior of Herceptin-loaded hydrogels revealed significant potential for preventing the recurrence of HER2+ breast tumors following breast-conserving surgery.

Tumors are intricate formations composed of not only cancer cells but also stromal cells, including tumor-associated fibroblasts and macrophages [202]. In addition to altering the structure and composition of extracellular matrix-mimicking hydrogels, efforts have been directed at enhancing cellular variety in 3D hydrogel culture models to more accurately reflect cancer cell responses to treatment. A new immune-enhanced tumor organoid system was created by Shelkey et al. They encapsulated a murine 4T1 TNBC cell line and mouse spleen-derived immune cells in hydrogel based on methacrylated collagen and thiolated hyaluronic acid. The study followed the impact of bacterial metabolites from species found in the immunomodulatory host microbiome on the immune checkpoint blockade response. These metabolites significantly increased the induction of cancer cell apoptosis. The results of the study showed a synergistic effect of the immunomodulatory host microbiome analog found in bacteria and immune checkpoint blockade therapy in the induced apoptosis of tumor cells. The hydrogel-based immune-enhanced 3D tumor organoid model proved to be a powerful platform for precision therapeutic testing [203]. Advancements in hydrogel models have enabled the development of ex vivo and patient-derived scaffolds that better capture the heterogeneity of breast cancer tissues, their various subtypes, and their complex responses to treatment, offering a more accurate representation compared to traditional single- or double-cell line models. The potential of some hydrogels was explored by Koch et al. in a study conducted in an ex vivo culture model. They tested heparin hydrogels functionalized with star-shaped PEG and maleimide using human mammary tissues. The results showed that the structural support provided by the hydrogels maintained the viability of human tissues for up to 3 weeks, although the preservation of the epithelial phenotype and hormone receptors was observed for up to 2 weeks [204]. Patient-derived organoid models developed for therapeutic testing purposes highlight the potential of bioengineered hydrogel platforms in replicating native tumor-like 3D cancer biology. This advance could revolutionize drug screening practices and increase the efficacy of therapeutic treatments. However, many limitations remain. Performing multidrug tests in patient-derived organoid system cultures depends on tumor samples extracted by central needle biopsy that may contain only a small number of cancer, stromal, and immune cells. In this case, the final result cannot be conclusive. Sometimes, the hydrogels currently used for patient-derived organoid system cultures do not match the extracellular matrix of the native tissue or the tumor formation environment of a patient. Although organoid models hold great potential for precision medicine testing, continued development and optimization are still needed, and several challenges need to be addressed before they can be widely applied in clinical settings to help real-world patients.

On the other hand, an ongoing challenge remains in finding a long-term solution for filling the deficiencies caused by partial mastectomies, which are often performed following a breast cancer diagnosis. Currently, many available methods for addressing these defects are temporary and require multiple repeated procedures. Sometimes, these approaches fail to offer a permanent solution, leaving patients to endure ongoing interventions and the associated physical and emotional toll. As a result, there is a pressing need for more effective, lasting treatments that can adequately address both the functional and aesthetic concerns of individuals who undergo partial mastectomies. New injectable porous hydrogel structures, created from natural polymers like gelatin and alginate, have been developed for use in breast reconstruction and regeneration following or even during partial mastectomy [205]. The hydrogels demonstrated remarkable mechanical and physical characteristics, with compressive and tensile modulus values comparable to those of native breast tissue. In vitro cell viability tests conducted on mouse preadipocytes revealed strong biocompatibility. The minimally invasive nature of this method, combined with the exceptional qualities of the scaffold, enables the filling of complex gaps, such as some deep deformities or those resulting from mastectomies, while reducing surgical costs and significantly enhancing patient well-being.

## 4. Challenges and Limitations

### 4.1. Mechanical Strength and Durability Issues

While hydrogels offer exceptional biocompatibility, water retention, and drug delivery capabilities, their mechanical strength and durability remain major challenges in emergency therapy applications. Hydrogels are primarily composed of water and polymer networks, making them inherently soft and weak under mechanical stress and limiting their use in load-bearing or high-stress environments. They often lack sufficient toughness to withstand stretching, compression, or friction. The softness and low modulus of hydrogels, derived from their significant water content, mean that they have low mechanical strength compared to stiffer materials such as metals, ceramics, or even some synthetic polymers. This softness can limit their ability to resist stress, deformation, and deformation under mechanical loading or during movement. In clinical applications, such as wound dressings, tissue scaffolds, and implantable devices, hydrogels must maintain structural integrity under stress. This limits their use in applications such as wound dressings or those requiring structural support, such as bone or cartilage regeneration or implantable devices. The mechanical properties of hydrogels depend largely on the degree and nature of crosslinking within their polymer network. Achieving an optimal balance between crosslinking and flexibility can be difficult to achieve, as inadequate mechanical strength can lead to failure in tissue regeneration, and stiffness can lead to complications such as inflammation or tissue rejection. Repeated stretching or compression can cause structural failure. This is a critical issue for injectable and wound-healing hydrogels, which must remain intact during movement.

Potential solutions are hybrid hydrogels incorporating reinforcing agents (e.g., nanofibers, graphene, or synthetic polymers) that enhance strength. While increasing strength, hydrogels must maintain flexibility for biological applications. Overly stiff hydrogels may lose biocompatibility, affecting cell migration and healing. A solution for balancing strength and flexibility is the use of double-network hydrogels combining two interpenetrating polymer networks, using adaptive crosslinking increasing both toughness and elasticity and hydrogel blends combining rigid and soft polymers for optimal mechanical properties. Moreover, self-healing hydrogels feature dynamic bonds that can repair themselves after mechanical damage, and crosslinked networks using covalent and ionic crosslinking improve resilience against tearing.

Hydrogels in high-motion areas (e.g., joint or skin applications) degrade faster due to friction and mechanical stress. Prolonged use leads to a loss of function and reduced efficacy over time. Wear resistance can be enhanced by toughened coatings, additional protective layers, hydrogel composites integrating elastomers or nanomaterials with increased durability, and bioinspired hydrogels mimicking natural resilient materials like tendons or cartilage. The mechanical strength and durability of hydrogels can be affected by environmental factors. Hydrogels degrade quickly under extreme conditions, such as high temperatures, acidic or enzymatic environments, or in the presence of other chemical species in the environment. This can limit their effectiveness in infected wounds or long-term implants. Their lifespan in harsh environments can be increased by the controlled degradation of hydrogels that adapt to pH, temperature, or enzymatic changes. Shear and tensile strength under dynamic conditions are important in hydrogels used in clinical applications, which are often subjected to dynamic mechanical loading, especially when used in soft tissue repair, joint stabilization, or implantable devices. Many hydrogels do not have the necessary shear and tensile strength to maintain their structural integrity under these conditions.

Hydrogels’ mechanical limitations pose challenges in emergency therapy, but innovative material engineering is addressing these issues. Hybrid, self-healing, and reinforced hydrogels are emerging solutions that enhance durability without compromising biocompatibility and functionality. As research advances, next-generation hydrogels will become stronger, more resilient, and better suited for high-stress medical applications.

### 4.2. Risk of Infection and Biodegradation Control in Hydrogel-Based Emergency Therapy

Hydrogels are susceptible to infection risks and uncontrolled biodegradation, which can compromise their effectiveness. Addressing these challenges is crucial for ensuring safe and long-lasting hydrogel applications in emergency care.

Hydrogels can degrade through several mechanisms, depending on their composition and structure. In general, the primary degradation mechanisms in hydrogels are as follows:

(i) Hydrolytic degradation: This occurs by breaking the polymer chains due to reactions with water molecules. For hydrogels made from natural or synthetic polymers with hydrolysable bonds, water can trigger the gradual cleavage of these bonds, leading to degradation of the material. Hydrolytic degradation is generally specific to biocompatible hydrogels, such as those made from polyesters or polysaccharides.
(ii) Enzymatic degradation: In hydrogels derived from natural polymers such as collagen, chitosan, or hyaluronic acid, enzymes present in the body (such as proteases, lipases or cellulases) can break down the polymer network. This degradation process is often slower and more specific, providing a controlled decomposition of the material according to the body’s natural enzymatic environment.
(iii) Oxidative degradation: Some hydrogels, especially those containing certain synthetic polymers or crosslinkers, are susceptible to oxidation when exposed to reactive oxygen species (ROS), which are generated during inflammation or oxidative stress. This can lead to the breakdown of the polymer matrix and damage the structural integrity of the hydrogel.
(iv) Physical degradation: In some cases, hydrogel degradation can occur due to physical processes such as swelling, compression, or mechanical stress. Over time, the hydrogel can lose its shape or structure, leading to a gradual degradation of the material.

In addition, the rate at which hydrogels biodegrade can vary significantly depending on several factors, such as the following:

(i) Polymer composition: Natural polymers, such as alginate or collagen, tend to degrade faster than synthetic polymers, such as polyethylene glycol (PEG) or polyvinyl alcohol (PVA), which may be more resistant to degradation.
(ii) Crosslinking density: The degree of crosslinking in a hydrogel influences its stability. Highly crosslinked hydrogels generally degrade more slowly because the interconnected polymer chains are more resistant to breakage. Conversely, hydrogels with low crosslinking densities degrade more rapidly.
(iii) Environmental conditions: The pH, temperature, and ionic strength of the environment can affect the rate of degradation. For example, acidic conditions can accelerate hydrolytic degradation, while high temperatures or oxidative conditions can increase the rate of decomposition of certain materials.
(iv) Body fluids and enzymes: The presence of specific enzymes or body fluids (e.g., blood, lymph) can influence the rate of degradation, especially for hydrogels designed to degrade in response to specific biochemical cues. Enzyme-mediated biodegradation is often slower but more controlled, offering the potential for prolonged function in the body.

Some hydrogels degrade too quickly, reducing their therapeutic effectiveness. If degradation is too fast, drug-loaded hydrogels may fail to deliver medication over the intended period. Moreover, the breakdown of certain synthetic hydrogels may release harmful residues, causing inflammation. On the other hand, the incomplete degradation of hydrogels, requiring surgical removal, is not ideal in emergency therapy. It is imperative to adjust the degradation rate for different applications: long-lasting hydrogels for implants and slow-release drug delivery, fast-degrading hydrogels for temporary wound dressings, and hemostatic agents. Strategies for controlled biodegradation include the use of biodegradable crosslinkers that allow for predictable and controlled breakdown, developing enzyme-responsive hydrogels that degrade only in specific biological conditions, designing hydrogels that degrade in response to body temperature or pH, and hybrid hydrogels with adjustable degradation rates combining natural (e.g., collagen, alginate) and synthetic polymers for optimized biodegradation.

Despite their advantages, the use of hydrogels in emergency medical settings may lead to several potential side effects that need to be carefully considered.

(i) Infection risk: The high water content of hydrogels creates a favorable environment for bacterial and fungal growth. Hydrogels used for chronic wounds, burns, or implants may become contaminated over time. Many hydrogels lack intrinsic antimicrobial activity, increasing the risk of biofilm formation. Cross-contamination by improper handling or storage can introduce pathogenic bacteria, leading to secondary infections. Infection risks can be reduced by incorporating antimicrobial agents like silver nanoparticles, chitosan, or iodine to inhibit bacterial growth. Smart hydrogels for infection monitoring and pH-sensitive hydrogels can detect early signs of infection and release antimicrobial agents when infection is detected. Pre-sterilized, single-use hydrogel dressings for emergency wound care and improved storage solutions can prevent microbial contamination before application.
(ii) Inflammation and immune response: Certain degradation products, especially those from synthetic hydrogels, can induce localized inflammation or an immune response. This can cause pain, redness, and swelling at the site of application and could complicate the healing process, especially if the hydrogel degradation products are perceived as foreign by the immune system.
(iii) Toxicity induced by degradation products: The breakdown of certain hydrogels, especially synthetic ones, can produce toxic byproducts that can harm surrounding tissues. For example, the degradation of polyesters or polyurethanes can release acidic byproducts, which can lead to tissue irritation, necrosis, or delayed healing.
(iv) Mechanical failure: Some hydrogels, especially those used for structural support or as scaffolds in emergency care, can degrade too quickly, compromising their mechanical properties and leading to failure in their function. For example, a hydrogel used in wound healing or burn care may lose its structural integrity too early, leading to reduced efficacy and potential complications.
(v) Bioaccumulation: In cases where synthetic polymers are used, degradation products can accumulate in the body over time if not properly removed. This could lead to chronic toxicity or long-term adverse effects, especially if the degradation of the hydrogel is not well controlled.

To optimize the use of hydrogels in emergency medical applications, various strategies are being explored to manage degradation rates and mitigate side effects:

- Controlled degradation—Tailoring the chemical composition and crosslinking of hydrogels can allow for better control over their degradation rates, for example, allowing them to degrade at specific rates in response to physiological conditions such as changes in pH or the presence of certain enzymes.
- Use of biodegradable polymers—The development of hydrogels based on fully biodegradable natural polymers (e.g., collagen, hyaluronic acid) or biocompatible synthetic materials (e.g., PLGA—poly(lactic-co-glycolic acid)) can lead to non-toxic byproducts that are safely absorbed or eliminated by the body.
- The addition of biocompatible additives such as anti-inflammatory agents, antimicrobial agents, or antioxidants to the hydrogel formulation can reduce the risk of infection, inflammation, or oxidative damage during the hydrogel degradation process.

In conclusion, while hydrogels offer immense potential in emergency medical use, their degradation mechanisms, rates, and potential side effects need to be carefully evaluated and controlled to ensure optimal performance and safety. Continuing the development of hydrogels with controlled degradation profiles and minimal side effects will pave the way for their wider and more effective use in emergency and critical care settings.

Addressing these issues through antimicrobial agents, smart sensors, and controlled degradation strategies can significantly improve hydrogel safety, effectiveness, and patient outcomes. Future research on nanotechnology, bioengineering, and responsive materials will further enhance infection resistance and biodegradation predictability, making hydrogels more reliable in emergency medical applications.

### 4.3. Cost and Scalability of Hydrogel-Based Medical Applications in Emergency Medicine

Hydrogels have significant potential for use in emergency medicine, offering a range of benefits such as wound care, hemostasis, drug delivery, and tissue repair. However, cost and scalability remain key barriers to making hydrogel-based treatments more accessible and practical, particularly in emergency settings where rapid, cost-effective solutions are needed.

Hydrogels often involve complex synthesis processes that contribute to high production costs, including raw material and additive (such as antimicrobial agents or growth factors) costs. Tailored hydrogel formulations to meet the specific needs of different medical applications (e.g., wound dressings vs. drug delivery systems) can lead to higher development costs. The need for precise control over crosslinking, porosity, and drug encapsulation makes large-scale production technically challenging. Cleanroom environments and sterilization processes needed for medical-grade hydrogels increase costs. The need of streamlined synthesis techniques requires research into simpler, more cost-effective hydrogel fabrication methods. Increasing the availability of bio-based polymers, the sustainable production of raw materials, and the implementation of automated hydrogel production processes can enhance scalability and drive down costs. Compact, pre-packaged, portable hydrogel kits for emergency care that are easy to distribute and use in field conditions would improve access to hydrogel treatments in disaster zones.

Scaling hydrogel production from laboratory or small-scale settings to commercial levels presents several hurdles: (i) reproducibility: maintaining batch-to-batch consistency in the properties of hydrogels, such as swelling behavior, mechanical strength, and drug release profiles, can be difficult when scaling up; (ii) quality control: ensuring strict quality control in large-scale production is essential for medical-grade products, variations in crosslinking density, molecular weight, or pore size can significantly impact hydrogel performance. Developing industry standards and protocols for hydrogel production can help achieve consistency at a larger scale. Scalable hydrogel synthesis platforms utilizing continuous-flow reactors and high-throughput technologies can improve scalability. Advancements in 3D printing technologies may allow for the on-demand production of hydrogels in specific shapes and sizes, especially for implants or custom treatments.

Even if hydrogel production costs are reduced, there are still significant costs involved in clinical adoption and implementation. Hydrogel-based medical devices require extensive testing, clinical trials, and regulatory approval, which add substantial costs. For new hydrogel treatments, especially those used for wound healing or tissue regeneration, significant long-term clinical data are needed to prove efficacy and safety, which can delay adoption and increase costs. In many cases, cost-effectiveness is crucial for gaining insurance coverage reimbursement for hydrogel treatments. Hydrogel-based solutions may be too expensive for widespread use in low-income or resource-limited regions, where emergency medical supplies are often scarce. This is especially concerning for acute wound care and disaster relief, where affordable, readily available solutions are critical. Creating locally sourced, simple hydrogel formulations (e.g., based on natural polymers like agarose or gelatin) could help lower costs. Bio-based and renewable materials such as seaweed-derived alginate or plant-based cellulose offer promising options for making hydrogels affordable in low-resource settings. Moreover, hydrogels often require specific storage conditions (e.g., temperature control, moisture control) to maintain their effectiveness. This can complicate logistics, particularly in emergency response scenarios, where quick deployment is essential. Developing stable, long-lasting hydrogel formulations that do not require stringent storage conditions can increase practicality in emergency settings. Some hydrogel dressings can be freeze-dried or lyophilized for easier transportation and storage. In conclusion, challenges with scalability and cost-effective clinical implementation must be addressed through advanced manufacturing methods, scalable production platforms, and collaborative efforts with healthcare systems for the widespread adoption of hydrogel-based therapies in emergency medicine.

### 4.4. The Gap Between Laboratory Research and Practical Deployments

The disparity between laboratory research and real-world clinical applications presents a significant challenge, especially when it comes to translating promising innovations into effective and practical solutions in emergency care. While laboratory studies provide valuable information and controlled conditions to test new materials or technologies, real-world clinical environments are much more complex, with a number of unpredictable variables, such as patient diversity, environmental factors, logistical constraints, and emergency care in critical situations. Although there are many promising results of hydrogels obtained in laboratory studies, especially in the areas of wound healing, drug delivery, and tissue regeneration, there are numerous obstacles that prevent their smooth transition from research to real-world clinical applications: the complexity of human physiology, in vivo behavior, physiological stress, consistency and reproducibility, manufacturing constraints, regulatory approval, and safety concerns.

## 5. Future Perspectives and Innovations

Future advancements will focus on enhancing the functionality, adaptability, and scalability of hydrogels to improve their effectiveness in life-saving situations, including the following aspects:

- *Smart and stimuli-responsive hydrogels* will be designed to respond dynamically to physiological and environmental changes, allowing for precision medicine in emergency care: pH-responsive hydrogels that release drugs in response to infection-induced acidity changes; temperature-sensitive hydrogels that solidify or liquefy based on body temperature, aiding in burn and trauma treatment; enzyme-activated hydrogels that trigger drug release only when specific enzymes indicate infection or injury; and light- and magnet-responsive hydrogels that allow remote-controlled drug delivery for non-invasive treatment. These innovations will enable real-time, site-specific therapy, reducing treatment time and side effects in emergencies.
- *Injectable and self-healing hydrogels* administered via syringes that solidify inside wounds or internal injuries, preventing excessive bleeding, repairing themselves after damage, and ensuring long-term stability in tissue engineering and wound closure. These properties will revolutionize minimally invasive treatments, especially in prehospital and battlefield care.
- *Hydrogel-based wearable and implantable devices*. The integration of hydrogels with wearable biosensors and implantable devices will enable continuous health monitoring and automated therapy: hydrogel biosensors that detect changes in glucose, lactate, or inflammation levels, enabling timely drug release and implantable drug-eluting hydrogels that offer long-term medication release, reducing the need for repeated dosing in critical conditions. These advancements will support automated, patient-specific treatment approaches in emergency medicine.
- *Nanotechnology-enhanced hydrogels* incorporating nanoparticles with hydrogels will enhance their strength, conductivity, and drug-carrying capacity, enabling controlled drug delivery and antimicrobial properties, beneficial for burns and chronic wounds. Such innovations will help create stronger, longer-lasting, and highly efficient medical hydrogels for emergency care.
- *AI-integrated and 3D-printed hydrogels*. Artificial intelligence (AI) and 3D printing will transform hydrogel-based emergency solutions, allowing for personalized and on-demand production. AI-designed hydrogels will optimize formulations for specific emergency applications. Moreover, 3D and 4D bioprinted hydrogels will enable customized wound dressings and tissue scaffolds for rapid organ repair. These developments will make hydrogels more adaptable, efficient, and widely accessible. AI could improve the functionality and properties of hydrogels in particular:

- In the design and optimization of materials by using machine learning (ML), algorithms can optimize the synthesis of hydrogels and predict how different material compositions and processing conditions affect the final properties of hydrogels (e.g., mechanical strength, degradation rate, and bioactivity). In addition, the ability to rapidly analyze large amounts of data and predict optimal material combinations can significantly accelerate the development of next-generation hydrogels. AI can also help design hydrogels with controlled-release capabilities for drug delivery in cases where the polymer network needs to degrade at a specific rate or respond to environmental stimuli such as pH or temperature.
- AI and computational models can simulate how hydrogels might perform in complex biological environments. This helps researchers predict how the material will behave over time, including how it interacts with cells, tissues, or drugs. This predictive capability reduces the need for expensive and time-consuming experiments.
- Three-dimensionally printed hydrogels can also be used to produce patient-specific implants or prosthetics. These implants can be designed to integrate with biological tissues and provide targeted drug delivery or act as tissue substitutes. AI can further improve design by predicting the best material properties and design features based on the anatomy and clinical needs of the individual patient.

However, batch-to-batch variability in 3D printing and the complexities involved in scaling AI algorithms to design hydrogels for large-scale production pose a challenge. Therefore, ensuring consistent material quality and reproducibility is essential for clinical applications, especially when working with biologically sensitive products such as tissue scaffolds or drug delivery systems.

- *Sustainable and cost-effective hydrogels*. To increase scalability and affordability, future hydrogels will incorporate eco-friendly and biodegradable materials; plant-based hydrogels derived from cellulose, alginate, and chitosan, ensuring biocompatibility and sustainability; and low-cost crosslinking techniques that reduce manufacturing costs, making hydrogels more accessible for low-resource settings. These advancements will ensure that high-quality emergency treatments are available to a wider global population.
- *Recent advances in hydrogels commercialization.* Over the past decade, advances in hydrogel research have accelerated, leading to significant advances in commercialization and clinical trial development. These advances are driven by both improvements in hydrogel material properties, the development of 3D printing technologies, and the integration of AI. Despite these advances, the clinical use and commercialization of hydrogels on a large scale presents numerous challenges. The commercialization of hydrogels involves both the development of new hydrogel formulations and the expansion of manufacturing techniques to meet market demands. To heal wounds faster and reduce pain and scarring, several types of hydrogel-based wound care products are marketed:

- Hydrocolloid dressings are widely useful in clinical settings especially for chronic wounds, such as diabetic ulcers or pressure ulcers, developed by companies such as Smith & Nephew and Convatec.
- Bioactive hydrogels for tissue regeneration containing bioactive agents, such as growth factors or peptides that promote tissue regeneration, are marketed by the companies MIMETAS and CELLINK, for example.
- Self-healing hydrogels for long-term wound management are developed by the company 3M.
- Injectable hydrogels to deliver drugs or biological substances directly to a targeted site, reducing systemic side effects, are marketed by the companies Medytox and Evolus.
- Hydrogel microneedles, an atraumatic microtechnique to deliver vaccines or drugs, are marketed by Microneedle Technologies.

In conclusion, the future of hydrogel-based emergency therapy lies in smart, self-healing, AI-powered, and sustainable solutions. By combining nanotechnology, AI, and 3D bioprinting, next-generation hydrogels will revolutionize emergency medicine, offering faster, more efficient, and personalized treatments for critical conditions.

## Figures and Tables

**Table 1:** Conventional versus hydrogel treatments in emergency care.

Conventional Treatments	Common Benefits	Features of Hydrogel-Based Materials
Hemostatic dressings (gauze, bandages, and adhesive dressings) Tourniquets Saline or antiseptic irrigation Antibiotics Splints and casts Opioid medications (e.g., morphine) or NSAIDs (e.g., ibuprofen) to manage pain in cases of trauma or burns	Rapid patient stabilization and management Life-saving Weight-based dosing of medications Pain relief	Ability to rapidly activate within seconds of application. Ability to stop moderate to severe bleeding for a wide range of wound types and surfaces. Sufficient adhesion strength and mechanical properties, even under wet and dynamic conditions, to effectively seal wounds and prevent material removal from the bleeding site. Strong adhesion and mechanical stability in wet and dynamic environments to securely seal wounds and prevent detachment. Potential to not interfere with any metabolic pathway that could lead to substantial physiological complications. Easy storage, long shelf life, and stability for extended usability. Excellent antimicrobial properties to prevent infections at the wound site. Non-toxic, biocompatible, and hemocompatible. Biodegradability aligned with the natural wound-healing process. Hydrogel dressings provide cooling effects, directly reducing pain.

## Data Availability

Not applicable.
